# Liquid Refractive Index Measurement System Based on Electrowetting Lens

**DOI:** 10.3390/mi10080515

**Published:** 2019-08-01

**Authors:** Shi-Long Li, Zhong-Quan Nie, Yan-Ting Tian, Chao Liu

**Affiliations:** 1 School of Instrumentation and Optoelectronic Engineering, Beihang University, Beijing 100191, China; 2 College of Physics and Optoelectronics, Taiyuan University of Technology, Taiyuan 030024, China; 3 Beijing Advanced Innovation Center for Big Data-based Precision Medicine, Beihang University, Beijing 100191, China

**Keywords:** refractive index measurement, electrowetting, liquid lens

## Abstract

In this paper, a liquid refractive index (LRI) measurement system based on an electrowetting lens was proposed. The system is composed of a light source, a collimating lens, a liquid measurement chamber (LMC), an electrowetting lens and an image sensor, which is integrated into a cylindrical cavity. The refractive index of the LMC changes with the addition of the measured liquid, and the incident light cannot be focused on the image plane. By adjusting the driving voltage of the electrowetting lens, the curvature of the liquid-liquid interface changes to focus the incident light onto the image plane. The refractive index of the liquid could be measured according to the voltage value. The proposed LRI measurement system has no mechanical moving parts, and the imaging surface remains stationary, which can make the measurement simply and correctly. The experiments show that the refractive index measurement range of the system can be turned from ~1.3300 to ~1.4040, and the measurement accuracy is 10^−4^. The system can be used to measure the optical properties of liquids and has broad potential applications in chemical reagent detection and pharmaceutical testing.

## 1. Introduction

A refractive index is an important optical parameter of liquids. With the refractive index, the optical properties, the purity, dispersion and diffusion coefficient of the fluids can be analyzed [1,2]. Conventional liquid refractive index (LRI) measurement systems can be classified into refractometers and interferometers, according to measurement methods [3,4,5,6]. The principle of the refractometer is based on the phenomenon of refraction or reflection generated by light passing through the surface of the liquid. Then the refractive index is measured according to the law of refraction or reflection. The grazing incidence method and laser irradiation method [7] are the typical methods. These measurement methods are relatively simple. However, the liquid in the measurement system is usually in contact with air, and is not suitable for measuring volatile and toxic liquids [7,8,9,10,11]. The most common refractometer is the Abbe refractometer (AR) [8], which is based upon the principle of total reflection. The advantages of AR are high precision, and it can read the value of the refractive index directly from the dial screen. However, AR has a large volume and complex operation. The interferometer employs the wave property of light to interfere or diffract with the determinant in order to measure the refractive index. Representative measurement methods are fiber, Young’s interferometry [9] and CCD measurement methods [10]. Unfortunately, the instruments based on the wave measurement methods are generally complicated. The operating system is usually cumbersome, and it also needs more liquids usage [7,8,9,10,11]. Thus, it is not suitable for the measurement of precious liquids.

Based on the above discussion, an LRI measurement system, which is economical, simple to operate, accurate in measurement, and not limited by the characteristics of the liquid, is highly desired.

In recent years, liquid optical devices have attracted the wide attention of researchers because of the unique advantages of small size, low cost, fast response time and high light transmittance. Some methods for measuring the refractive index of liquids using optical flow control devices have also been studied [12,13,14,15,16,17]. Among them, liquid lenses are widely used in measurement systems, due to the performance of adaptive zoom than the conventional solid lenses, such as the adaptive optical zoom system and the imaging system [18,19,20,21,22,23,24,25]. As far as we know, there are currently only two research groups that use liquid lenses to measure the refractive index. In 2012, Calixto et al. proposed a refractive index measurement through image analysis with an optofluidic lens [26]. The test solution is injected into a hollow lens made of Polydimethylsiloxane (PDMS). The light passing through the hollow lens forms a bright line on the image surface. The LRI can be tested by analyzing the visibility of the image. However, the hollow lens made of PDMS has not designed an inlet or outlet, which is not conducive to measure the refractive index of the liquid continuously. Moreover, the texture of PDMS is soft, and can be deformed or broken easily. In 2014, Cheng et al. also proposed a refractive index measurement by prism auto collimation [27], which can measure the refractive index of solids and liquids. However, the measurement system is complicated by the use of more optical components.

In this paper, a novel LRI measurement system based on an electrowetting lens was proposed. When different measured liquids are injected into the measuring cavity, the image planes will show the unfocused light spots. By adjusting the external driving voltage of the electrowetting lens, the spot will focus on the image plane, and the refractive index of the liquid can be calculated according to the applied voltage value. Compared with the previous works, the liquid measurement chamber (LMC) in the proposed system is small in volume and sealed by a cavity. Thus, both a precious liquid and a volatile liquid can be measured. Besides, the electrowetting lens has the advantages of small volume and adjustable focus. The proposed system does not need to move any mechanical component during the measurement. Moreover, the proposed system is simple to operate and can directly derive the LRI using the values of the driving voltage. In summary, the proposed system has the advantages of small size, high integration, convenient carrying, simple operation, no restriction on liquid characteristics, and no mechanical moving parts.

## 2. Structure and Operating Principle

### 2.1. Structure and Mechanism of the System

**Figure 1:**
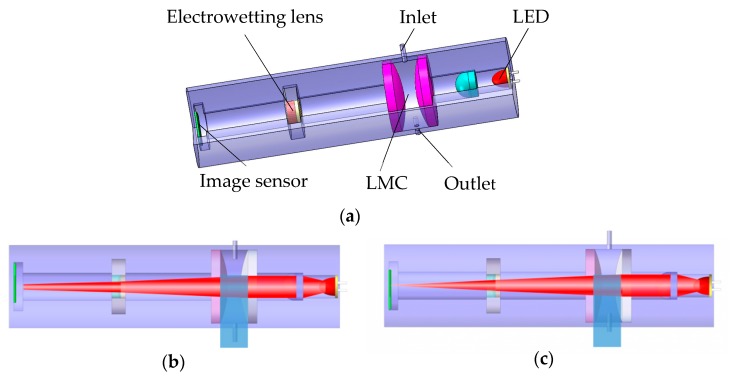
Structure and mechanism of the proposed system. (**a**) Cross-section of the system. (**b**) State of voltage off. (**c**) State of voltage on. (Where LMC is the liquid measurement chamber and LED refers to the light emitting diode).

Figure 1 shows the structure and mechanism of the proposed system. The light emitting diode (LED) light source, the collimating lens, the LMC, the electrowetting lens and the image sensor are assembled in a cavity, wherein the LMC has two channels for convenience to inject liquids and clean the cavity, as shown in Figure 1a. In the initial state, the measured liquid is injected into the LMC, and the LED light passing through the collimator lens becomes a parallel beam. The parallel beam is deflected by the LMC, and the deflected light passes through the electrowetting lens to reach the image sensor to form a divergent light spot. Then, the light is focused on the image plane by adjusting the drive voltage of the electrowetting lens, as shown in Figure 1b,c. We can get the curvature of the electrowetting lens by the voltage value, and calculate the LRI according to the ‘yun’ ray-tracing method.

### 2.2. Theory of the Proposed System

**Figure 2:**
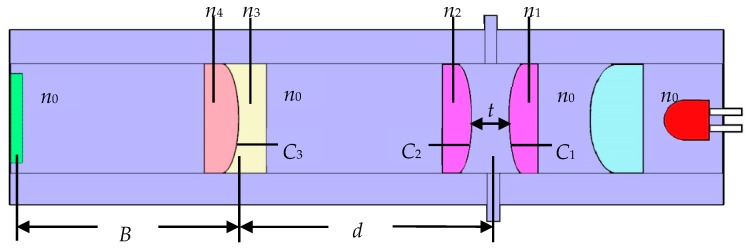
Mathematical model of the system.

As shown in Figure 2, the distance between the two optical lenses constituting the LMC is *t*, the refractive indices are *n*_1_ and *n*_2_, and the radii of curvature are *C*_1_ and *C*_2_, respectively. The refractive indices of the two filling liquids of the electrowetting lens are *n*_3_ and *n*_4_. The radius of curvature of the liquid contact surface is *C*_3_. The distance between the center of the liquid measurement chamber and the center of the electrowetting lens is *d*. The back focal length of the entire measurement system is *B*. According to the ‘yun’ ray-tracing method in geometric optics, it can be deduced that the liquid measurement cavity power *φ*_a_ and the electrowetting lens power *φ*_b_ can be calculated by Equations (1) and (2), respectively.

*φ*_a_ = 1/*f*_a_ = (*n*_x_ − *n*_1_) *C*_1_ + (*n*_2_ − *n*_x_) *C*_2_ − [(*n*_x_ − *n*_1_) (*n*_2_ − *n*_x_) *tC*_1_*C*_2_]/*n*_x_ (1)

*φ*_b_ = 1/*f*_b_ = (*n*_4_ − *n*_3_) *C*_3_ (2)

The back focal length of the entire measurement system is *B*, which can be expressed as:

*B* = (1 − *dφ*_a_)/(*φ*_a_ + *φ*_b_ − *φ*_a_*φ*_b_*d*) (3)

In summary, the refractive index *n*_x_ of the measured liquid is a function of the following parameters, namely:

*n*_x_ = *f* (*t*, *d*, *B*, *n*_1_, *n*_2_, *n*_3_, *n*_4_, *C*_1_, *C*_2_, *C*_3_) (4)

Among them, *C*_1_, *C*_2_, *n*_1_, *n*_2_, *n*_3_ and *n*_4_ are known quantities; *t*, *d* and *B* can be directly measured, *C*_3_ can be measured according to the curvature of the electrowetting lens and the values of the driving voltage. Through the above formulas, the refractive index of the measured liquid can be calculated.

### 2.3. Fabrication of the Proposed System

**Figure 3:**
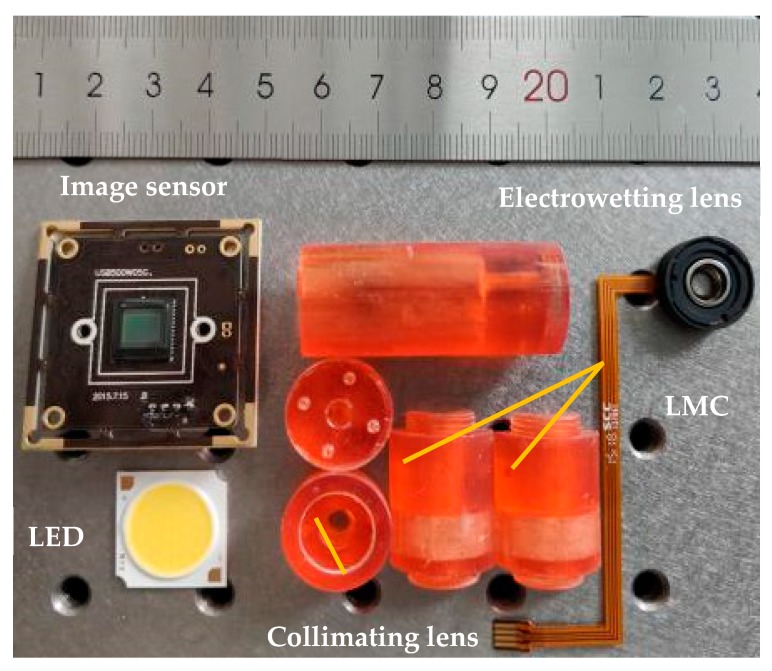
Components of the proposed system.

The cavity of the LRI measurement instrument and other implements are fabricated by a 3D printer. The outer diameter and the inner diameter of the cavity are 18 mm and 10 mm, respectively, and the length is about 70 mm. The inlet and outlet with the same diameter of 1 mm are symmetrically distributed on the LMC. The collimating lens is made of BK7 optical glass with a radius of curvature of 50 mm. The LMC consists of two flat-concave lenses. The lenses are made of K9 optical glass with a radius of curvature of 150 mm, the center thickness of each convex lens is 1.5 mm, and the distance between the two convex lenses is 4 mm. The electrowetting lens is an Arctic-39N0 developed by Corning Inc. (New York, NY, USA) The distances between the LED, the collimating lens, the liquid measurement chamber, the electrowetting lens and the image sensor are 7 mm, 10 mm, 25 mm, 25 mm, respectively. The components of the LRI measurement system are shown in Figure 3.

## 3. Experiments and Discussion

### 3.1. Experiment of the Proposed System

**Figure 4:**
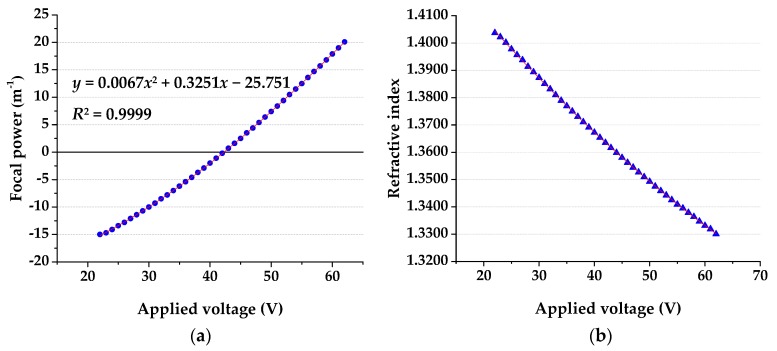
(**a**) Focal power of an electrowetting lens under different applied voltages. (**b**) Refractive index under different applied voltages.

The electrowetting lens is an essential component of the liquid refractive index meter system. It should be noted that the external driving voltage adjustment of the electrowetting lens is realized by a computer software operating a driving board connected to the computer. The values of the focal power corresponding to the different driving voltages of the electrowetting lens is shown in Figure 4a. At the same time, the relation is also fitted to the curve: *y* = 0.0067*x*^2^ + 0.3251*x* − 25.751 (*R*^2^ = 0.9999).

According to the formula in Section 2.2, the relationship between the driving voltage and the refractive index is calculated, as shown in Figure 4b. 

**Figure 5:**
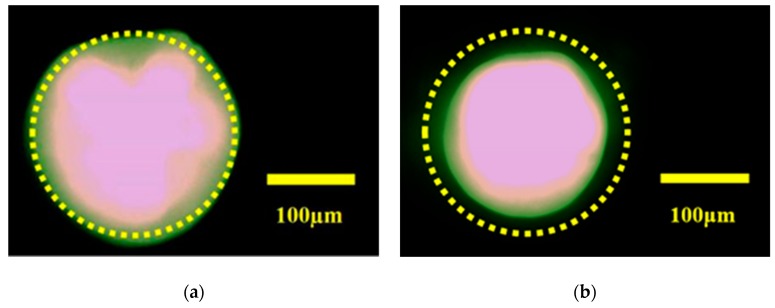
(**a**) Light spot under the state of voltage off. (**b**) Light spot under the state of voltage on.

We assembled the components and set up the measurement system according to Section 2.3, and measured the refractive index of a 10% NaCl solution. First, a 10% NaCl solution is injected into the LMC. The light from the LED passes through the collimating lens, the LMC and the electrowetting lens, and reaches the image plane to form a divergent spot, as shown in Figure 5a. Then, we adjust the applied driving voltage of the electrowetting lens. When the applied voltage is increased from 0 V to 49 V, the spot diameter gradually becomes smaller. When the driving voltage reaches 50 V, the spot becomes larger than before. Therefore, when the applied driving voltage is 49 V, the spot is minimized on the image plane, as shown in Figure 5b. According to the data of Figure 4b, the refractive index of 10% NaCl is 1.3509. Compared with the reported value 1.3515, the error is −0.0444. The experimental results show that the LRI measurement system can measure the refractive index of the liquid correctly, and its measurement accuracy is 10^−^^4^.

Based on the above experiments, the refractive indices of different concentrations of NaCl solution were measured according to the above experimental procedures. The measurement results are shown in Table 1. 

The experimental results show that the refractive index of the NaCl solution becomes larger as the concentration increases, and the applied driving voltages of the electrowetting lens decrease as the refractive indices increase. The average error of the theoretical and practical measurements is −0.0207.

Besides, we have characterized the proposed liquid refractive index measurement system using the Brix standard. The concentration of sucrose solutions was measured, as shown in Table 2. As can be seen, when the sucrose solution concentration is 42%, the applied driving voltage of the electrowetting lens reaches the limit value of 22 V. So, the system can measure the concentration of the sucrose solution in the range from ~0% to ~42%, that is, the range of Brix is ~0% to ~42%, and the corresponding refractive index range is ~1.3300 to ~1.4040.

To further analyze the error of the system, we also measured the refractive index of distilled water (DW), ethanol and isopropanol with a known refractive index. The measurement results and errors are shown in Table 3. From the measurement results, the proposed system can measure the refractive index of a liquid correctly.

According to the experimental results, the liquid refractive index (LRI) measurement system based on the electrowetting lens can provide a reliable method to measure the refractive index of liquid correctly, and its accuracy is 10^−4^.

### 3.2. Discussion

The key novelty of the LRI measurement system is that it can easily measure the LRI directly by adjusting the applied voltage without any moving mechanical parts. The system can be used within areas where it is necessary to measure the refractive index of liquids simply and quickly. However, some of the performance of this system can also be improved. As can be seen from Figure 4b, the LRI measurement range of the system herein is about 1.3300~1.4040, which is smaller than that of the conventional method using the Abbe refractometer (1.3300–1.7000). 

The reason may be that the LMC used in the system consists of two planoconvex lenses, which we can consider as cylindrical lenses. When a liquid with a broad refractive index is measured, the focal length of the cylindrical lens is significantly increased, thereby exceeding the zooming ability of the electrowetting lens. 

**Figure 6:**
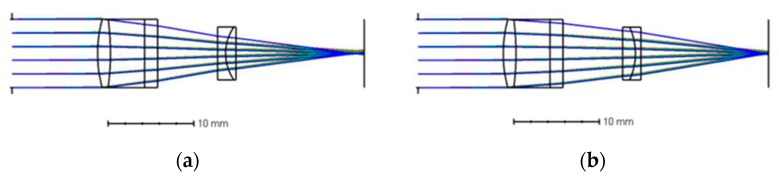
Structural simulation with different refractive indices. (**a**) Refractive index 1.3300 @ voltages of 62 V. (**b**) Refractive index 1.7000 @ voltages of 22 V.

To solve this issue, we can change the structure of the LMC to expand the range of the refractive index of the liquid. As shown in Figure 6, the LMC is composed of a convex lens and a flat glass, which are 4 mm apart and are made of K9 optical glass. The two surfaces of the convex lens have curvatures of 15 and −30, respectively. The center thickness of the convex lens is 1.5 mm. The thickness of the flat glass is 1.5 mm. Under this structure, the liquid measurement range can be extended to 1.3300–1.7000. 

Besides, the accuracy of the LRI measurement system proposed in this paper is 10^−4^, which is lower than the accuracy of the prism-based refractive index measurement system by 10^−6^. One of the reasons is that the applied driving voltage of the electrowetting lens is incremented by 1 V. The relationship between the driving voltage and the refractive index is nonlinear, which affects the measurement accuracy of the system and causes measurement errors. But if the drive voltage increment could be reduced to 0.5 V, the accuracy of the liquid refractive index would be significantly improved, and the measurement errors would also be reduced accordingly.

At last, another error source of the LRI measurement system was analyzed, and the corresponding solutions were proposed. When a plurality of sample liquids is continuously measured for their respective refractive indices, the fluids may remain in the LMC, so that mixing with different liquids may cause an inaccurate refractive index measurement. However, when processing the LMC, we can spin-coat a layer of Teflon hydrophobic coating on the inner wall which can significantly reduce the possibility of liquid residue in the LMC and improve the accuracy of the measurement. 

## 4. Conclusions

In this paper, an LRI measurement system based on an electrowetting lens was proposed. The system can measure the refractive index of different liquids continuously by adjusting the applied driving voltage of the electrowetting lens without moving any mechanical components. The proposed LRI measurement system has the advantages of simple operation, small size and portability. The system can be used to measure the optical properties of liquids, and has the potential to be widely used in the fields of the chemical and the food industries. Experimental results show that the measurement range of the system can be turned from ~1.3300 to ~1.4040, and the system measurement accuracy is 10^−4^. In later work, we will further optimize the structure of the system, expand the measurement range of the liquid refractive index, and improve the measurement accuracy. 

## Figures and Tables

**Table 1:** Refractive index and error measurement with different concentrations of NaCl solution.

Concentration of NaCl Solution (%)	Refractive Index in the Literature	Measured Refractive Index	Error (%)	Applied Voltage (V)
1	1.3347	1.3346	−0.0075	59
5	1.3428	1.3425	−0.0223	54
10	1.3515	1.3509	−0.0444	49
15	1.3603	1.3598	−0.0368	44
20	1.3709	1.3710	0.0073	38

**Table 2:** Refractive index with different concentrations of sucrose solution.

Sucrose Solution Concentration (%)	Measured Refractive Index	Applied Voltage (V)
5	1.3409	55
10	1.3475	51
20	1.3635	42
30	1.3831	32
40	1.4002	24
41	1.4022	23
42	1.4040	22

**Table 3:** Refractive index and error measured with different liquids.

Liquid	DW	Ethanol (50%)	Isopropanol	Alcohol
**Refractive index in the literature**	1.3330	1.3590	1.3750	1.3900
**Measured refractive index**	1.3330	1.3598	1.3749	1.3893
**Error (%)**	0.0098	0.0593	0.0001	0.0464
**Applied voltage (V)**	62	44	36	29
